# Predisposing Factors, Microbial Characteristics, and Clinical Outcome of Microbial Keratitis in a Tertiary Centre in Hong Kong: A 10-Year Experience

**DOI:** 10.1155/2015/769436

**Published:** 2015-06-18

**Authors:** Alex Lap-Ki Ng, Kelvin Kai-Wang To, Chile Chi-Lai Choi, Leonard Hsu Yuen, Suk-Ming Yim, Keith Shun-Kit Chan, Jimmy Shiu-Ming Lai, Ian Yat-Hin Wong

**Affiliations:** ^1^Department of Ophthalmology, LKS Faculty of Medicine, The University of Hong Kong, Pok Fu Lam, Hong Kong; ^2^Department of Microbiology, LKS Faculty of Medicine, The University of Hong Kong, Pok Fu Lam, Hong Kong; ^3^Department of Ophthalmology, Queen Mary Hospital, Pok Fu Lam, Hong Kong

## Abstract

*Purpose. *To study the risk factors, microbial profile, antibiotic susceptibility pattern, and outcome for microbial keratitis over the past 10 years in a tertiary center in Hong Kong.* Methods. *All cases with corneal scraping performed in Queen Mary Hospital, Hong Kong from January 2004 to December 2013 were included. Clinical outcome was defined as poor if the final visual acuity (VA) was abnormal or worse than presenting VA, a major complication occurred, or therapeutic keratoplasty was required.* Results. *347 scrapes were performed in the 10-year period growing 130 microorganisms (32.3% culture positive rate). Contact lens use was the commonest risk factor. The commonest isolates were coagulase-negative* Staphylococcus* and* Pseudomonas aeruginosa. *Fluoroquinolone susceptibility was tested in 47 Gram-negative bacteria with 93.6% susceptibility (100% for* Pseudomonas*). 90.7% of cases had good visual outcome. Multivariate logistic regression showed age (*p* = 0.03), trauma (*p* = 0.006), and ulcer size >3 mm (*p* = 0.039) to be independently associated with poor outcome.* Conclusion*. There was no shifting trend in the isolate distribution or emergence of resistant strains in our study. Contact lens wear was the commonest risk factor, with* Pseudomonas* being the most frequent isolate in this group. It remained 100% susceptible to fluoroquinolones and 97% cases had good visual outcome.

## 1. Background

Microbial keratitis is one of the commonest causes of corneal blindness worldwide. The mainstay of diagnosis is corneal scraping. However, the clinician has to decide on the antibiotic regime before the culture and antibiotic susceptibility results are available. This decision is often based on patient's demographics, risk factor profile, and the local microbial distribution pattern. As geographical and climatic factors result in regional differences in the pattern of microbial isolates, local epidemiologic studies are necessary [1]. The antibiotic susceptibility profile is also important in view of reports on the emergence of methicillin or fluoroquinolone resistance in North America and Asia in the past decade [2–5]. The in vivo resistance correlates with a worse clinical outcome as well [6].

In Hong Kong, the last large-scale epidemiological study on microbial keratitis was over 15 years ago [7]. The aim of this study was to investigate for any changes in the microbiological and antibiotic susceptibility pattern, risk factors profile, and clinical outcome in a Hong Kong tertiary referral center over the past 10 years.

## 2. Method

This retrospective study included all patients who received diagnostic corneal scraping in Queen Mary Hospital, Hong Kong, from January 2004 to December 2013. Corneal scrapings were performed with a sterile number 15 surgical blade under topical anesthesia. Samples were routinely inoculated onto chocolate, blood, and Sabouraud's agar plates and sent to the Department of Microbiology for culture and antibiotic susceptibility testing. Any positive growths from the specimens would be reported by the microbiologist. The antibiotic susceptibility was determined according to the CLSI (Clinical and Laboratory Stand Institute) criteria.

### 2.1. Data Collection

All culture and antibiotic susceptibility results obtained from the corneal scrapings were retrieved from the Department of Microbiology database. Cases notes were reviewed. Data collection included patient demographics, risk factors profile, and the clinical progress. Cases with incomplete clinical data were excluded from the analysis. These included cases that defaulted follow-up after initial presentation or with incomplete documentation of risk factors or progress in the clinical notes. Cases with absence of corneal infiltrates were also excluded from analysis.

Risk factors considered, including contact lens use, trauma, history of corneal and ocular surface disorders, and presence of systemic illness (which either resulted in immunocompromised state or poor self-care), were shown in Table 1. For clinical features, size and location of corneal ulcer and presence of hypopyon in the anterior chamber were recorded. Size of lesion was categorized as small if less than 3 mm or large if more than 3 mm. Presence of good initial response to antibiotic was defined as improvement in pain or reduction in size of ulcer or infiltrate in the first 48–72 hours. Clinical outcome was defined as good if the visual acuity at final visit was better than 0.7 (Snellen), or, in cases where there was poor premorbid visual acuity from preexisting ocular pathology, there should be no drop in final visual acuity as compared with presenting visual acuity. Poor outcome was defined if there was a drop in visual acuity or if a major complication occurred, which included corneal perforation, endophthalmitis, or had therapeutic keratoplasty performed.

### 2.2. Analysis

Statistical analysis was performed with SPSS version 21 (IBM). The number of each cultured isolate and antibiotic resistance pattern were tested with Spearman correlation for any changing trends over the 10-year period. The microbial pattern in different risk factor groups was compared with chi-square test. Univariate and multivariate analyses by logistic regression were used to examine for any risk factors affecting the outcome. The study received approval by the Institutional Research Ethics Board of the University of Hong Kong (reference number: UW14-043) and complied with the Declaration of Helsinki.

## 3. Results

347 corneal scrapings were performed in the 10-year period. 43.4% were males and 53.5% were females. Mean age was 46.2 ± 21.1 (range 10–96; median 46, interquartile range 27–63). The peak incidence occurred in the 21–30 subgroup (68 cases (19.6%), mean 25.1 ± 2.9) and the 51–60 subgroup (56 cases (16.1%), mean 54.6 ± 2.7). After exclusion, 260 cases were available for analysis of risk factors and 241 cases for clinical outcome (19 cases defaulted follow-up before healing of ulcer).

### 3.1. Culture Results

**Figure 1:**
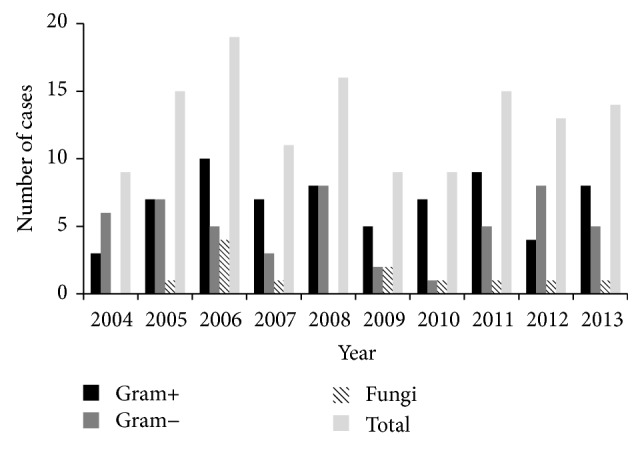
Distribution of Gram-positive, Gram-negative bacteria, and fungus over 10 years.

The distribution of microbial isolates was shown in Figure 1 and Table 2. 32.3% (112) scraping sample was culture positive and grew 130 microorganisms. 16 cases had polymicrobial growth. The most common bacterial growths were coagulase-negative* Staphylococcus*,* Pseudomonas aeruginosa*, and* Staphylococcus aureus*. Neither the Gram-positive nor the Gram-negative group demonstrated a shifting trend over the 10-year period (*p* = 0.634 and 0.722; *r* = 0.172 and −0.129, resp.).

### 3.2. Antibiotic Resistance

Fluoroquinolone susceptibility was tested in 47 Gram-negative bacteria with 93.6% susceptibility. Among 17 cases of* Staphylococcus aureus* and 25 cases of coagulase-negative* Staphylococcus* tested for methicillin resistance, there was 1 case (5.9%) of methicillin-resistant* Staphylococcus aureus* (*MRSA*) and 7 cases (28%) of methicillin-resistant coagulase-negative* Staphylococcus* (*MRCNS*). All of these were susceptible to vancomycin. No significant increasing trend in methicillin resistance was observed (*p* = 0.139; *r* = −0.502). 92 isolates were tested for gentamicin with 88.0% susceptibility. It was higher (93.3%) if we only considered Gram-negative bacteria (45 isolates). All* Pseudomonas* were 100% susceptible to fluoroquinolones, aminoglycosides, and ceftazidime.

### 3.3. Risk Factors

82.3% (214 cases) had at least 1 identifiable risk factor and 21.2% (55 cases) had multiple risk factors. The distribution of the risk factors was shown in Table 1. Contact lens wear was the most common, accounting for 42.7% (111 cases). The average age was 28.4 (range 11–69), with male : female ratio 1 : 2. 34.2% (38 cases) had positive culture and* Pseudomonas* accounted for half of the cultured bacteria*. Fusarium* was cultured in 3 cases. When compared with noncontact lens-related cases, contact lens-related keratitis grew a statistically significant higher proportion of Gram-negative bacteria (*p* < 0.0005). The second commonest risk factor was the presence of keratopathies or ocular surface disease, with 82 cases (31.5%). Average age was 58. 52.3% of these cases were culture positive and Gram-positive* cocci* were the commonest isolate (14 coagulase-negative* Staphylococcus* and 10* Staphylococcus aureus*), followed by* Pseudomonas* (7 cases). When compared with cases without keratopathies or ocular surface diseases, this group yielded significantly more Gram-positive growths (*p* = 0.007). 48 cases (18.5%) had either immunocompromised state or mental illness resulting in poor self-care. Many of these cases had multiple comorbidities. Average age was 62.4 years. 39.6% of these cases grew Gram-positive bacteria in which 16 cases were Gram-positive cocci. Eight cases (16.7%) grew Gram-negative bacteria with* Pseudomonas* predominance (7 cases, 15.6%). For traumatic cases, there were 27 cases (10.4%), with average age 40.3. Most were superficial injury causing corneal abrasion but with delayed presentation. 22.2% (6 cases) had positive bacterial culture and all were Gram-positive* cocci* with* Staphylococcus aureus* being the commonest.

### 3.4. Presenting Features

225 cases were available for analysis, as others were excluded due to poor documentation of lesion size owing to a transition to computerized record around 2008 in our center. 197 cases (87.6%) had initial corneal ulcer smaller than 3 mm while 28 cases (12.4%) were larger than 3 mm. Presence of hypopyon was found in 30 cases (13%).* Pseudomonas* was significantly associated with the presence of hypopyon on presentation (48.3% in* Pseudomonas* versus 13.5% in non-*Pseudomonas*, *p* < 0.0005, chi-square test).

### 3.5. Antibiotic Choice, Initial Response, and Clinical Outcome

91.5% of cases started topical fluoroquinolones as first line treatment. 38% of these cases were combined with aminoglycosides (gentamicin). 6.5% (17 cases) started with combination of fortified antibiotics (ceftazidime plus tobramycin or vancomycin). 3.5% (9 cases) received antifungal agents on top of initial antibacterial treatment (as these were referred as suspected fungal cases). Topical antifungal agents employed included topical natamycin and/or 0.1% amphotericin B. Systemic antifungal agents with voriconazole, ketoconazole, or fluconazole were used in 3 cases.

90% (239) of cases showed good initial response with improvement in either pain, infiltrate size, epithelial defect size, or amount of hypopyon. 12% (32) of cases needed to step up treatment, either due to lack of treatment response after 48–72 hours or as guided by the antibiotic susceptibility result of the corneal scrape.

90.7% had good outcome and 9.3% (24 cases) had poor outcome. Two cases developed endophthalmitis resulting in blind eye. One culture-negative case had corneal biopsy performed at a later stage which found* Acanthamoeba*. One initially culture-negative case required enucleation and histology revealed fungal infection. Therapeutic penetrating keratoplasty was performed in one case.

Logistic regression was performed to analyze where the clinical outcome was affected by the factors shown in Table 3. Older age group (average 62.7), history of trauma, and presenting lesion size >3 mm were found to be associated with poor outcome with both univariate (*p* = 0.05, 0.009, and 0.044, resp.) and multivariate (*p* = 0.03, 0.006, and 0.039, resp.) analyses.

## 4. Discussion

The overall positive culture rate in this series was 32.3%. When compared with other keratitis studies published in the past decade (Table 4), their culture growth rate of corneal scrapings ranged from 25.6% to 78% [1, 8, 10–12, 14–17, 21, 22]. Ours was among the lower yield range. One main reason was that our hospital was a regional tertiary center. Most cases that presented to us have already been started on topical antimicrobial therapy. Our center also received referral from private ophthalmologists, and this group of partially treated patients often has already received broad-spectrum antibiotics therapy, commonly topical levofloxacin or moxifloxacin. Another reason was the indication of performing corneal scraping was not limited to infectious keratitis. In some cases, the clinical diagnosis was noninfective, such as peripheral ulcerative keratitis or herpetic keratitis. Clinically there was absence of suppurative infiltrate. Nevertheless, corneal scraping was performed in order to exclude presence of secondary bacterial infection as this had important implication in the management plan. These cases, as expected, yielded negative culture result and contributed to the lower overall rate of positive growth.

We had a slightly higher proportion of Gram-positive than Gram-negative bacteria, with coagulase-negative* Staphylococcus* (*CNS*) and* Pseudomonas* being the most common growths, respectively. Most other studies in developed countries also had similar findings where there were a higher proportion of Gram-positive bacteria, except two studies from the United Kingdom [14, 16]. But, in the studies performed in Asian developing countries, they had more fungal keratitis as majority were traumatic keratitis [18–20]. On the other hand, in developed Asian regions, such as Shanghai, Beijing, Taiwan, Japan, and Hong Kong (current study), the findings resembled those of developed European and American regions, where the most common risk factors were contact lens use and fungal keratitis only consisted of a small portion of cases [3, 10, 21, 23]. This highlighted the fact that wide geographical variation existed even within the same continent, and the clinician should pay attention to the background and travel history of the patient especially in international cities like Hong Kong.

The Hong Kong Keratitis Study Group studied 223 cases in 1997 to 1998 [7]. The positive culture rate was 35%, which was very similar to our series. The main risk factors were history of ocular surface disease followed by trauma, and the most prevalent causative bacteria were* Pseudomonas*. Compared to them, our series had a higher portion of contact lens-related keratitis. The shift in the risk factor for keratitis could be due to an increasing number of teenagers and young patients using contact lens and with poor contact lens habit. This suggestion could be supported by another study that looked into 18 cases of pediatric keratitis (aged under 18) from 2001 to 2010 in Hong Kong [24]. In their series, contact lens use was the most common risk factor. In our study, 15% (17 patients) of the contact lens-keratitis subgroup were aged 18 or younger. In both their study and our contact lens-keratitis subgroup,* Pseudomonas* followed by coagulase-negative* Staphylococcus* was the most common pathogen. This showed consistency in the microbial pattern in contact lens-related keratitis in Hong Kong.* Pseudomonas* also remained fully susceptible to third-generation cephalosporin, aminoglycosides, and quinolone antibiotics in both studies, which spanned over 15 years. This was very reassuring as these were the most commonly used agents by ophthalmologists in Hong Kong, and no increase in resistant strains was reported.

In our study, fluoroquinolone susceptibility was tested in 47 Gram-negative bacteria with 93.6% susceptibility. When compared with a recent 10-year study in UK, they reported a 94.4% susceptibility to ciprofloxacin for their Gram-negative bacteria [16]. In another recent 11-year study in Toronto, they found a 97.4% susceptibility to ciprofloxacin for their Gram-negative isolates, with 97.8% for* Pseudomonas aeruginosa* [1]. Therefore, our fluoroquinolone susceptibility rate was actually quite similar to those reported by overseas studies. As for* Pseudomonas aeruginosa*, we found 100% susceptibility to fluoroquinolone in our series. The low resistant rate of Gram-negative bacteria in our series, including* Pseudomonas aeruginosa*, may be related to the relatively low usage of quinolone in general in Hong Kong. There were two main reasons for the low usage of quinolone. First of all, the most commonly prescribed antibiotic eye drops in our primary care setting was chloramphenicol instead of fluoroquinolones. Fluoroquinolone eye drops were mainly only prescribed by ophthalmologists and therefore abuse of these antibiotic agents was less of an issue. Secondly, in patients with systemic infections such as pneumonia, our physicians always try to avoid the use of quinolone as a first line agent. This is because of the high incidence of tuberculosis in Hong Kong, and the widespread use of fluoroquinolone is discouraged to prevent the emergence of quinolone-resistant tuberculosis. The zero resistance to fluoroquinolone in our* Pseudomonas* cases could also explain the good initial treatment response (90%) in our series. We would continue fluoroquinolone monotherapy as the initial first line therapy in most of our infectious keratitis cases. In fact, Shalchi et al. gave same recommendation in their 10-year review in United Kingdom [16]. A recent meta-analysis also supported the use of fluoroquinolone as the first choice for empirical treatment in most cases of suspected bacterial keratitis too [25].

Older age, larger ulcer size, and traumatic cases were associated with poor outcome with a loss of final visual acuity. The older age group patients often had coexisting keratopathies, ocular surface diseases, or systemic diseases. Other study also reported worse outcome in patients over 60 years old [9]. We would recommend antibiotic with broad Gram-positive coverage in older patients since we found a significantly higher proportion of Gram-positive isolates in both presence of keratopathy and presence of systemic conditions. Larger lesion size (>3 mm) was also associated with poor outcome, as this group of patients often had delayed presentation or factitious pathogens, although we found no correlation of microorganisms with the ulcer size. We would recommend admission for fortified antibiotic for these patients to ensure a better compliance to therapy and closer monitoring.

*Limitations*. This study was limited by its retrospective design. The decision of performing corneal scraping in clinically bacterial keratitis cases was not unified. Some early cases with small lesions (<1 mm) were treated empirically without obtaining corneal scraping. As a result, there was very likely an underreporting of the number of bacterial keratitis cases and cultured isolates. The study was also carried out in a tertiary referral centre and therefore was treating a higher portion of more difficult or treatment-refractory cases. The number of underlying risk factors might have been overlooked by the attending clinician as well. For example, the presence of keratopathies or systemic illness might not have been performed consistently in a retrospective study. For the antibiotic susceptibility testing, different groups of bacteria were tested with different sets of antibiotics. For example, fluoroquinolones were not routinely tested in Gram-positive isolates. Therefore, we could not directly compare the percentage of fluoroquinolone resistance with other studies. Regarding the clinical outcome, we could only rely on parameters such as final visual acuity and cases with complications. The exact number of days for complete resolution of infiltrate and reepithelialization could not be determined as the follow-up intervals varied among different clinicians and in different cases.

## 5. Conclusions

This study was the largest case series of consecutive corneal scrapings in Hong Kong. It identified contact lens wear as the most common risk factor with* Pseudomonas* being the most prevalent pathogen in this group. Its susceptibility to most first line antibiotic agents remained 100% throughout the decade, and monotherapy with fluoroquinolones was an effective first line treatment in most of our cases. More aggressive treatment approach should be employed in keratitis belonging to the older age group, larger presenting ulcer size, and traumatic cases in view of their association with a poorer outcome.

## Figures and Tables

**Table 1:** Risk factors for microbial keratitis.

Risk factors	Number of cases^1^	Most common microbial isolate
(1) Contact lens use	111	*Pseudomonas *

(2) Ocular trauma	27	*Staphylococcus aureus *

(3) Presence of keratopathy or ocular surface disease	82	Coagulase-negative *Staphylococcus *
(i) Presence of corneal scars or irregular ocular surface	19 (23.2%)	
(ii) History of corneal surgical procedures	11 (13.4%)	
(iii) Exposure keratopathy	10 (12.2%)	
(iv) Bullous keratopathy	7 (8.5%)	
(v) Superimposed infection following an epithelial defect	7 (8.5%)	
(vi) Superimposed infection following active herpetic keratitis	6 (7.3%)	
(vii) Dry eyes with corneal involvement	5 (6.1%)	
(viii) Neurotrophic keratopathy	3 (3.7%)	
(ix) Others	13 (15.9%)	

(4) Systemic conditions^2^	48^2^	Coagulase-negative *Staphylococcus *
Immunocompromised state		
(i) Diabetes mellitus	25 (52.1%)	
(ii) End-organ failure	8 (16.7%)	
(iii) Use of immunosuppressant including steroids	7 (14.6%)	
(iv) Terminal malignancies	3 (6.3%)	
Mental illness with poor self-care^3^	17 (35.4%)	

^1^214 cases (82.3%) had at least 1 risk factor identified. 55 cases (21.2%) had more than 1 risk factor.

^2^Some cases had multiple conditions.

^3^This group included moderate-to-severe mental retardation, moderate-to-severe dementia, and schizophrenia with self-neglect.

**Table 2:** Distribution of microbial isolates.

	Number of isolates	Percentage (%)
Gram-positive bacteria:		
Coagulase-negative* Staphylococcus *	34	50.0
*Staphylococcus aureus *	17	25.0
Other Gram-positive cocci^*∗*^	7	10.3
Other Gram-positive rods^#^	8	11.8
Other Gram-positive bacteria^∧^	2	2.9
Total	**68**	**100.0**

Gram-negative Bacteria:		
*Pseudomonas *	33	66
Enterobacteriaceae^†^	8	16
Other Gram-negative bacteria^‡^	9	18
Total	**50 **	**100**

Fungi:		
*Fusarium *	4	33.3
*Candida *	3	25
Others^*∗∗*^	5	41.7
Total	**12**	**100**

Note: 16 cases were polymicrobial cases.

^*∗*^Gram-positive cocci include other *Streptococcus* and *Staphylococcus* strains.

^#^Gram-positive rods include *Bacillus, Corynebacterium,* and *Diphtheroid*.

^∧^Other Gram-positive include *Micrococcus*.

^†^Enterobacteriaceae include *Escherichia coli, Klebsiella, Serratia, and Citrobacter*.

^‡^Other Gram-negative bacteria include *Moraxella, *Flavobacteria*, Acinetobacter,*and* Propionibacterium*.

^*∗∗*^Others include *Aspergillus, Penicillium,* and* Rhodotorula*.

**Table 3:** Univariate logistic regression of risk factors, presenting features and isolates with the clinical outcome. Age, traumatic etiology, and ulcer size >3 mm predicted a poor clinical outcome.

Variables	*p* value
Gender	0.447
Age	** 0.050**
Contact lens use	0.889
Trauma	** 0.009**
History of keratopathy or corneal surgery	0.123
Systemic immunocompromised state or mental illness with poor self-care	0.924
Initial response to antimicrobial therapy	0.503
Presence of hypopyon	0.990
Size of ulcer^*∗*^	** 0.044**
Bacterial isolate^*∗∗*^	0.245

^*∗*^Size of ulcer: classified into large (>3 mm) and small (<3 mm).

^*∗∗*^Divided into 5 groups for analysis: no growth; Gram-positive cocci; *Pseudomonas*; other microbial agents; polymicrobial cases.

**Table 4:** Comparison of current study results with other similar epidemiological studies.

Author	Place	Studied year	Total number of scrapes	Positive culture rate (%)	Distribution of isolates	Commonest isolate (descending order)	Other significant findings
Alexandrakis et al. (2000) [4]	South Florida, USA	1990–1998 (9 years)	2920	50% (1468)	Gram+: 48% Gram−: 50%	(i) *Pseudomonas* (ii) *Staphylococcus aureus *	(i) Increasing fluoroquinolone resistance of *Staphylococcus aureus* keratitis (ii) Increasing prevalence of *Staphylococcus Aureus* (iii) Decreasing prevalence of *Pseudomonas* (iv) Main risk factor: contact lens (*Pseudomonas* and *Serratia *most common in this subgroup)

Bourcier et al. (2003) [8]	Paris, France	January 1998–September 1999 (21 months)	300	68% (201)	Gram+: 83% Gram−: 17%	(i) Coagulase-negative*Staphylococcus * (ii) * Proprionibacterium acnes* (iii) * Pseudomonas*	(i) Gram-negative bacteria associated with more severe anterior chamber inflammation and larger infiltrate (ii) Main risk factor: contact lens: 50.2%; keratopathy: 21%; trauma 15% (iii) Coagulase-negative*Staphylococcus *most common in contact lens-keratitis (iv) Outcome: 99% respond to treatment; 5% very poor outcome

Butler et al. (2005) [9]	Sydney, Australia	September 1998–December 2002 (4 years 3 months)	190	62.8% (119)	Gram+: 75% Gram−: 25%	(i) Coagulase-negative *Staphylococcus * (ii) *Staphylococcus aureus* (iii) *Pseudomonas *	(i) **All cases >60 years old** (average age: 75.5) (ii) Local risk factors: 93.7%; systemic risk factors: 27.9% (iii) 7.9% HSV PCR+, which was associated with more perforation or severe thinning (80% versus overall 36%) (iv) Outcome: 17.9% PKP; 8.9% evisceration

Toshida et al. (2007) [10]	Japan	1999–2003 (5 years)	123	58.5% (72)	Gram+: 77.8% Gram−: 18.2% Fungi: 6.1% *Acanthamoeba*: 1.0%	(i) *Staphylococcus* (ii) *Streptococcus* (iii) *Corynebacterium *	Main risk factor: contact lens 54.5%; ocular surface disease 20.5%; previous ocular surgery 13.1%

Green et al. (2008) [11]	Australia	1999–2004 (5 years)	253	65% (164)	—	(i) *Pseudomonas* (ii) Coagulase-negative *Staphylococcus* (ii) *Staphylococcus aureus *	(i) 98% sensitivity to ciprofloxacin (ii) 100% sensitivity to gentamicin (Gram-negative) (iii) Main risk factor: contact lens 22%; ocular surface disease 18%; trauma 16%; prior surgery 11% (iv) Ocular surface disease associated with more severe keratitis (v) *Fusarium, Pseudomonas,* and Gram-negative organisms associated with more severe keratitis at time of scraping

Zhang et al. (2008) [3]	Beijing, China	2001–2004 (4 years)	1985	14.06% (279)	Gram+: 42.7% Gram−: 35.1%	(i) *Pseudomonas* (ii) *Corynebacterium * (iii) *Staphylococcus epidermidis *	Increasing ciprofloxacin resistance in Gram-positive cocci & Gram-negative bacilli

Ibrahim et al. (2009) [12]	Portsmouth, UK	1997–2003 (7 years)	1254	63.8% (800)	Gram+: 71.1% Gram−: 28.9%	(i) *Staphylococcus epidermidis * (ii) *Pseudomonas* (iii) *Staphylococcus aureus *	(i) Main risk factor: contact lens (ii) Outcome: 1.9% poor visual outcome

Edwards et al. (2009) [13]	Melbourne, Australia	May 2001–April 2003 (2 years)	88	78% (69)	—	*Staphylococcus species *	(i) **Only studied contact lens-related keratitis** (ii) Outcome: contact lens wearer age group of 15–24 had increased risk

Saeed et al. (2009) [14]	Ireland	September 2001–August 2003 (2 years)	90	36.6% (33)	Gram+: 33.3% Gram−: 54.5%	(i) *Pseudomonas* (ii) Coagulase-negative* Staphylococcus* (iii) *Staphylococcus aureus* (iv) *Streptococcus pneumonia *	(i) All studied cases were severe cases with hospital admission (ii) Main risk factor: contact lens 41.1%; anterior segment disease 21.1%; trauma (14%)

Cariello et al. (2011) [15]	Sao Paulo, Brazil	July 1975–September 2007 (>30 years)	6804	48.6% (3307)	Bacterial 81.6% +: 71.2% −: 27.0%	(i) Coagulase-negative* Staphylococcus* (ii) *Staphylococcus aureus* (iii) *Pseudomonas *	Main risk factors: corneal surgery, contact lens, and ocular trauma

Shalchi et al. (2011) [16]	East Kent, UK	1999–December 2009 (10 years)	476	34.2% (163)	Gram+: 38.9% Gram−: 61.1%	(i) *Pseudomonas * (ii) *Staphylococcus* *aureus* (iii) Coagulase-negative *Staphylococcus *	(i) Increase of Gram-negative isolates with time (ii) Main risk factor: contact lens (iii) Antibiotic sensitivity: (a) High sensitivity to combination of gentamicin and cefuroxime, as well as ciprofloxacin. (b) Trend of increasing resistance to chloramphenicol (c) Gram-positive fluoroquinolone resistance remained

Pandita and Murphy (2011) [17]	Waikato, New Zealand	January 2003–December 2007 (5 years)	265	65.6% (174)	Gram+: 78.2% Gram−: 20.2%	(i) Coagulase-negative *Staphylococcus* (40.8%) (ii) *Staphylococcus aureus* (11.5%) (iii) *Moraxella* (8.0%) (iv) *Pseudomonas *	(i) Fluoroquinolone: 99% sensitivity (ii) Tobramycin: Gram-positive: 95.5% sensitivity; Gram-negative: 100% (iii) Recommends fluoroquinolone monotherapy

Lichtinger et al. (2012) [1]	Toronto	2000–2011 (11 years)	1701	57.4% (976)	Gram+: 76.3% Gram−: 23.7%	(i) Coagulase-negative *Staphylococcus* (ii) *Staphylococcus aureus* (iii) *Pseudomonas *	(i) Decreasing trend in Gram-positive isolate (ii) Increasing methicillin resistance (29.1% of Gram-positive isolates) (iii) 43.1% of coagulase-negative *Staphylococcus* were multiple resistant

Tananuvat et al. (2012) [18]	Northern Thailand	April 2003–March 2006 (3 years)	310	25.6% (79)	Bacterial 49.3% Fungal 46.3%	(i) *Pseudomonas* (ii) * Fusarium *	(i) Main risk factor: 43.9% trauma; contact lens 3.5% (ii) Outcome: (a) Large ulcer >6 mm poor outcome (b) 41% required surgical procedure, commonest scleral patch graft

Tewari et al. (2012) [19]	Ahmedaba, India	July 2007–June 2008 (1 year)	150	59.3% (89)	Bacterial 65.1% +: 60.3% −: 39.7% Fungal: 34.9%	(i) * Staphylococcus* (ii) Coagulase-negative *Staphylococci * (iii) *Pseudomonas *	(i) *Aspergillis* most common fungi, followed by *Fusarium* (ii) Main risk factor: 90% trauma

Dhakhwa et al. (2012) [20]	Western Nepal	2007 (1 year)	414	72.5% (300)	Bacterial: 29.2% Fungal: 33.3%	(i) *Fusarium* (ii) *Staphylococcus epidermidis *	(i) Main risk factor: 33.3% trauma (ii) Outcome: 87.7% completely healed

Hong et al. (2013) [21]	Shanghai, China	January 2005–December 2010 (6 years)	1042	41.8% (436)	Gram+: 50% Gram−: 46.3%	(i) *Streptococcus species* (ii) *Pseudomonas *	(i) 8.3% methicillin-resistant *Staphylococcus aureus* (MRSA) (ii) 53.1% multiple resistant coagulase-negative *Staphylococci * (iii) Increasing fluoroquinolone resistance found

Ng (current study)	Hong Kong	January 2004–December 2013	347	32.3% (112)	Bacterial 90.8% +: 57.6% −: 42.4% Fungal 9.2%	(i) Coagulase-negative *Staphylococcus* (ii) *Pseudomonas* (iii) *Staphylococcus aureus *	(i) Contact lens-related keratitis most common (ii) No shifting trend in antibiotic resistance (iii) Poor outcome associated with age, size of ulcer >3 mm, and trauma etiology
